# A bioinspired peptide scaffold with high antibiotic activity and low *in vivo* toxicity

**DOI:** 10.1038/srep10558

**Published:** 2015-05-29

**Authors:** Francesc Rabanal, Ariadna Grau-Campistany, Xavier Vila-Farrés, Javier Gonzalez-Linares, Miquel Borràs, Jordi Vila, Angeles Manresa, Yolanda Cajal

**Affiliations:** 1 Department of Organic Chemistry, Faculty of Chemistry, University of Barcelona; 2 Department of Physical Chemistry, Faculty of Pharmacy, University of Barcelona; 3 Laboratory of Microbiology, Faculty of Pharmacy, University of Barcelona; 4 Barcelona Centre for International Health Research (CRESIB), Hospital Clínic – University of Barcelona; 5 Experimental Toxicology and Ecotoxicology Unit /CERETOX, Barcelona Science Park

## Abstract

Bacterial resistance to almost all available antibiotics is an important public health issue. A major goal in antimicrobial drug discovery is the generation of new chemicals capable of killing pathogens with high selectivity, particularly multi-drug-resistant ones. Here we report the design, preparation and activity of new compounds based on a tunable, chemically accessible and upscalable lipopeptide scaffold amenable to suitable hit-to-lead development. Such compounds could become therapeutic candidates and future antibiotics available on the market. The compounds are cyclic, contain two D-amino acids for *in vivo* stability and their structures are reminiscent of other cyclic disulfide-containing peptides available on the market. The optimized compounds prove to be highly active against clinically relevant Gram-negative and Gram-positive bacteria. *In vitro* and *in vivo* tests show the low toxicity of the compounds. Their antimicrobial activity against resistant and multidrug-resistant bacteria is at the membrane level, although other targets may also be involved depending on the bacterial strain.

Infectious diseases are the second most common cause of death worldwide. While multidrug resistance is observed in both Gram-positive and Gram-negative bacteria, two thirds of deaths are due to infection by Gram-negative bacteria^1^. Antibiotic-resistant bacteria have emerged as a major problem, especially for hospital-acquired infections, and the economic cost of multi-drug-resistant bacterial infections is immense. Of the 2 million people infected in the EU every year, some 175,000 (about 9%) die^2^. The Centre for Disease Control and Prevention (CDC) in Atlanta similarly estimates that in the USA, more than 2 million patients acquire antibiotic-resistant infections every year, of who at least 23,000 die^3^. Currently, more people die in USA of methicillin‐resistant *Staphylococcus aureus* than of AIDS. Meanwhile, the development of effective antibiotics has slowed down. Only seven new antibiotics were approved by the FDA from 2003 to 2012 (only two new classes since 1998), most of them effective against Gram-positive bacteria^4^,^5^,^6^,^7^,^8^,^9^,^10^,^11^,^12^.

All this makes it clear that there is an urgent need to develop new antibiotics against bacteria that are prone to acquire resistance to the antibiotics currently in use. The Infectious Disease Society of America has issued a proposal to develop new antibacterial drugs by 2020 against “ESKAPE” bacteria, which include the Gram-negative *Pseudomonas aeruginosa*, *Acinetobacter baumannii* and *Enterobacteriaceae* (i.e. *Escherichia coli* and *Klebsiella pneumoniae*), and the Gram-positive *Enterococcus faecium* and *Staphylococcus aureus*^13^. Furthermore, in 2012 the Innovative Medicines Initiative (IMI, co-funded by the European Union and EFPIA pharmaceutical companies) launched a €223 million New Drugs 4 Bad Bugs (ND4BB) program to spur the development of new antibiotics^5^,^14^,^15^. A major goal in antimicrobial drug discovery is the generation of new compounds capable of selectively killing resistant pathogens, possibly by novel mechanisms of action that could impede the development of resistance in the microorganisms.

Natural antimicrobial peptides (AMPs) have become a new class of antibiotics used to treat resistant bacterial infections including septic-shock^16^,^17^,^18^,^19^,^20^,^21^,^22^,^23^,^24^,^25^,^26^,^27^,^28^. They are a unique and diverse group of molecules produced by a variety of plant, invertebrate and higher animal species. Despite the diversity of their structures, most of them share common features, such as an amphipathic character and several charged residues. Similar structural features have been successfully induced in biodegradable antimicrobial polymers and nanostructures^29^. It has been claimed that the use of antibacterial agents that disrupt the membrane bilayer is a promising alternative approach to regular antibiotic action, since such agents have the additional advantage of being active against dormant bacteria (with slow or even no growth). Such metabolically inactive organisms can survive high concentrations of classic antibiotics, and extended treatment is therefore required for drug efficacy^30^.

Altogether it is evident that there is a clear unmet medical need in the field of infectious diseases and new, broad-spectrum and safer antibiotics are urgently required, particularly to fight extended and pandrug-resistant bacteria.

## Results and Discussion

### Design of compounds

Inspired by natural cyclic AMPs, particularly polymyxins, we embarked on a project to design cyclic lipopeptides with a chemically accessible scaffold structure, high activity and low toxicity. Since polymyxins are selective for Gram-negative bacteria, we also wished to explore features that could lead to potential activity against Gram-positive bacteria, particularly *S. aureus*.

**Figure 1:**
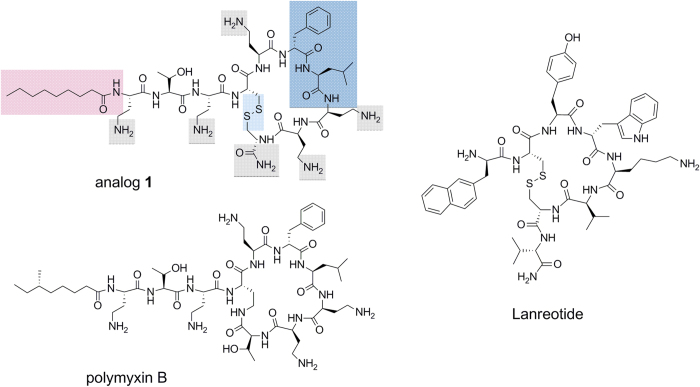
General structure of the cyclolipopeptide analogs (upper left, analog **1**, see Table 1). The structural and chemical features discussed for the design of the analogs are highlighted. Natural polymyxin B is shown below. The structure of lanreotide (right), a commercially available disulfide cyclic peptide having two D-amino acids is shown for comparison. Lanreotide is used for the treatment of acromegaly and neuroendocrine tumours and some 100-200 Kg are produced every year worldwide.

From the structural point of view, polymyxin B (PxB) and polymyxin E (PxE, colistin) are lipopeptide macrolactams with a tail-to-side-chain amide bond (Fig. 1), while the fatty acid moiety typically contains a stereocenter. Although the preparation of natural polymyxins via different synthetic routes has been reported, these two structural features would presumably complicate the synthesis of multiple analogs and the large-scale preparation of multigram (or kg) amounts for future clinical and therapeutic applications. One of our first objectives was therefore to reduce the complexity of the macrocyclic backbone scaffold. A plausible approach would consist of substituting the amide bond by an isosteric and more chemically accessible link, such as a disulfide bond. Disulfides are also chemical linkages present in some cyclic peptide drugs, as discussed below. Loop structures linked by disulfide bonds are not uncommon in cyclic AMPs. Examples are found in bactenicin (from cattle), lactoferricin, brevinins (from the frog *Rana brevipoda*), ranalexin (from the frog *Rana catesbeiana*), esculentin (from the frog *Rana esculenta*) and pipinin (from the frog *Rana pipiens*). The similarity between the macrocyclic heptapeptide structure in ranalexin and polymyxin was already noted by Zasloff^31^.

Similarly, Porro *et al.* described all-L-amino acid polymyxin-derived cyclic heptapeptides capable of binding to lipid-A with high affinity and detoxifying bacterial lipopolysaccharide (LPS) *in vitro*, but which showed no antibiotic activity^32^.

Altogether, this background and these structural data support the theoretical feasibility of substituting the tail-to-side-chain amide bond by a disulfide bond. This modification would imply changing both Thr^10^ and Dab^4^ for cysteines. In addition, the C-terminal cysteine would need to be derivatized as a carboxamide to mimic the neutral hydroxyethyl threonine moiety being substituted, as shown in Fig. 1.

Another important issue is the stereochemistry of the cysteines. Cysteine in position 4 should have the l configuration, whereas cysteine 10 ought to have the opposite d configuration, since the latter residue would be cyclized by means of the thiol side chain rather than from the backbone as in natural polymyxin (the Thr^10^ carboxy group). In the generation of analogues, both configurations were tested, but the most active compounds were those with a terminal D-cysteine amide.

Stability in the biological milieu was also taken into account when considering the design of compounds. Peptide drugs such as octreotide, lanreotide or vapreotide (analogs of somatostatin that consist of a disulfide cyclic octapeptide) were analyzed. Somatostatin is a natural hormone with a half-life of 2–3 minutes which is quickly proteolyzed when administered *in vivo*. However, lanreotide and vapreotide are much more stable to metabolic degradation (half-lives of around 2–3 hours) mainly due to the presence of D-amino acids (D-naphthylalanine/D-phenylalanine, and D-tryptophan). This suggests that the presence of D-amino acids in our disulfide-cyclized lipopeptides could improve their proteolytic stability and hence increase their half-lives and bioavailability. The most promising compounds we found in this respect contained two D-amino acids (the naturally occurring D-Phe^6^ and D-Cys^10^), as discussed below.

The disulfide bond may have additional potential as a pharmacological tool. On the one hand, it is known that PxB tends to accumulate over long periods (at least 48 h) in kidneys long after iv administration and after it has become barely detectable in serum (i.e., more than 30 times the serum concentration after 6 h). This suggests a selective uptake process in renal cells. Moreover, accumulation of PxB in kidneys could be correlated to its nephrotoxicity potential^33^,^34^. On the other hand, if this is the case with our novel PxB analogs, eventual uptake by renal cells would presumably reduce the disulfide and open up the peptide cycle due to the reducing intracellular environment (reduced glutathione and oxidorreductases) and thus facilitate the proteolysis of the linear sequence^35^,^36^,^37^. Hence, the disulfide link may provide PxB analogs with sufficient stability to reach the infectious target *in vivo*, whereas upon eventual accumulation and uptake by renal cells, reduction and subsequent decyclization would facilitate peptide proteolysis and potentially lower renal toxicity.

Finally, to further simplify the lipopeptide structure, the branched natural fatty acid tail was substituted by a linear one (nonanoic, decanoic or dodecanoic acids).

With all these structural features and a clear scaffold established, analogs were generated (Table 1). The initial idea consisted of introducing conservative substitutions of amino acids that are common in broad-spectrum AMPs, such as arginine, lysine and tryptophan. Hence, basic L-α,γ-diaminobutyric acid (Dab) residues were substituted by arginine, lysine, and also by ornithine or L-α,γ-diaminopropionic acid (Dap) residues (see also Supplementary Table S1). In particular, it was hypothesized that the guanidine group in arginine would increase electrostatic interactions with the phosphate groups of LPS due to its ambident nature and thus render higher antibiotic activity. Hydrophobic 6–7 positions were probed with other aromatic or aliphatic amino acids (D-tryptophan, norleucine and D-leucine). Threonine was maintained in position 2, as this amino acid seems to be involved in hydrogen bonding with the amide nitrogen of Dab^3^ and the carbonyl of Dab^4^, thereby helping polymyxin to bend and adopt a conformation necessary to interact with LPS^38^.

### Preparation and Evaluation of compounds

A first series of lipopeptides were synthesized by solid-phase chemical methods, cyclized at high dilution, purified (>95%) and their minimal inhibitory concentration (MIC) evaluated. A selection of the most representative compounds is shown in Table 1 (the other MIC values are in the Supplementary Table S1).

The first compound (**1**) consisted of the canonical application of our design principles to PxB. Thr^10^ and Dab^4^ were substituted by cysteines and the natural fatty acid was changed to the linear nonanoic acid. Moderate activity was obtained against representative Gram-negative bacteria, such as *P. aeruginosa* and *E. coli* (MIC 8 and 4 μg/mL, respectively). However, no activity was observed against Gram-positive *S. aureus*.

Addition of a single arginine did not improve this activity (analogs **2**, **3** and **4**). However, the introduction of two or three arginines (analogs **5**, **6** and **8**) conferred moderate activity against *S. aureus*. Additional arginines had a detrimental effect, particularly against Gram-negative bacteria (analogs **10** and **12**). Furthermore, cytotoxicity was found to increase with the number of arginines (Table 2). Hence, analogs with few (1 or 2 arginines) or no arginines were preferable from this point of view.

The introduction of D-cysteinamide at the C-terminal (**13**) also had positive effects, as activity against *E. coli* and *P. aeruginosa* was maintained, while the spectrum of activity apparently broadened towards *S. aureus*. We also explored the use of longer fatty acid moieties, (**36**, **37**) such as the decanoic acid (the fatty acid present in daptomycin) and dodecanoic acid, mainly to try to improve activity against Gram-positive bacteria, as described previously in the literature^38^,^39^,^40^.

Leucine, which contains a branched isobutyl side chain, was substituted with an isomeric norleucine, which has a linear butyl side chain. The idea behind this substitution was to introduce a more flexible and longer C4 hydrocarbon side chain, as in the linear fatty acid of the N-terminus, but essentially maintaining the lipophilicity of the residue.

Finally, when all these structural elements (decanoic/dodecanoic fatty acid, D-cysteinamide, norleucine, few arginines, or better, all-Dab analogs) were added together, activity against Gram-negative bacteria reached MIC values comparable to those of natural PxB (analogs **38** and **39**). Furthermore, activity against *S. aureus* reached values of 2-4 μg/mL. It is worth noting that both peptides **38** and **39** shared the same peptide sequence, with only a difference in the length of the N-terminal fatty acid.

To further explore the extent of the activity against Gram-positive bacteria of the most active sequence (all-Dab, Nle^7^, DCys^10^), its MIC values were evaluated in strains of *S. aureus* and *Enterococcus faecalis*, including clinical isolates and laboratory-derived strains at Cubist Pharmaceuticals (Lexington, MA). Lipopeptide **39** was chosen for this, as it is known that long fatty acids tend to favor activity against Gram-positive bacteria in polymyxin analogs^38^,^39^,^40^,^41^. The MIC values obtained demonstrated that its antibiotic activity was similar to that of daptomycin and vancomycin (see Table 3). In summary, new broad-spectrum antimicrobial lipopeptides were achieved.

### Activity against resistant and multi-drug-resistant Gram-negative bacteria

To study the potential activity of the most active sequences (compounds **38** and **39**), MIC assays were carried out with clinical isolates from resistant/multi-drug-resistant Gram-negative bacteria. Strains of *E. coli* and *P. aeruginosa* with resistance to quinolones and carbapenems as well as multi-drug-resistant bacteria were tested. The MIC results showed that the compounds were even more active against the selected strains than against regular ATCC bacteria strains, with many MIC values in the submicromolar range (0.5 μg/mL), which are comparable to those of natural PxB and colistin. It is worth mentioning that an *E. coli* strain containing the New Delhi metallo-beta-lactamase gene (NDM-1) isolated from a traveler to India^42^, which is resistant to β-lactamic antibiotics (penicillins, cephalosporins, monobactams, and carbapenems), aminoglycosides, and quinolones, showed MIC values for the new compounds of 0.5 μg/mL (see Table 4).

### *In vivo* acute toxicity test

An *in vivo* acute toxicity test was performed on CD-1 mice. The lethal dose (LD50) was determined according to the up-and-down procedure. Compound **38** was administered subcutaneously at designated doses (100, 200 and 400 mg/kg). Mice treated with 100 and 200 mg/kg of compound **38** survived with no signs of toxicity; whereas mice administered 400 mg/kg died. After 14 days, necropsy of the three surviving mice (dosed at 200 mg/kg) showed no signs of pathology in vital organs. LD50 was determined using the maximum likelihood method and a value of 283 mg/kg was obtained. This value is significantly higher (almost 5 times) than the LD50 reported for subcutaneous administration of PxB (59.5 mg/kg). Hence, analog **38** has a significantly lower toxicity than natural PxB, according to this toxicity test.

### Flow cytometry

The mechanism of action at the membrane level of our best candidate was studied using flow cytometry. To assess membrane permeability, a propidium iodide (PI) probe was used^43^,^44^. The fluorescence conferred by this probe is generally associated with cells that have lost their membrane integrity. The effect on membrane potential was evaluated with bis-(1,3-dibutylbarbituric acid) trimethine oxonol (BOX). Membrane depolarization leads to the incorporation of this anionic dye into the cell where it binds to lipid-rich intracellular compounds, resulting in an increase in fluorescence.

*E. coli* and *S. aureus* were used as models for Gram-negative and Gram-positive bacteria, respectively. PxB is known to target the membrane of Gram-negative bacteria, and was used as a control in *E. coli*. As expected, the flow cytometric analysis showed that PxB induces high levels of permeabilization quickly (around 70% in 120 min, see Table 5) and to a lesser extent, depolarization. However, depolarization seems to happen at the initial stages, since samples taken after 5 minutes of incubation showed only 11% permeability compared to around 45% depolarization (see supplementary Table S3 and supplementary Figure S4). These results suggest that the first effect of the presence of PxB in the medium is the collapse of the membrane potential (depolarization) which is followed by insertion and disruption of the cytoplasmic membrane (permeability).

In order to study the extent to which the dye is inserted into the bacteria, and thus characterize the insertion of PxB, a control experiment was also carried out with PxB nonapeptide (PxBN), a delipidated and truncated PxB derivative. PxBN is devoid of antibiotic activity but retains the capacity to bind to LPS and disorganize the outer membrane of Gram-negative bacteria. We hypothesized that in the case of bacteria treated with PxBN, PI would not cross the bacterial cytoplasmic membrane, as PxBN is only capable of disrupting the outer membrane. Our results confirmed this hypothesis, since no depolarization or permeabilization was detected (only a 3%-6% background) after 60 minutes of incubation at 5 μg/mL (10 times the MIC of PxB). A final control of staining consisting of bacteria cells subjected to a thermal shock (30 min, 70 °C) was also performed.

When *E. coli* (see Table 5 and supplementary Figure S4) was incubated with peptide **38**, a progressive, time-dependent permeabilization took place, but more slowly (7% in 30 min; 43% in 120 min) than with natural PxB. However, dissipation of the membrane potential occurred very quickly: around 50% in 30 min. According to the results, it seems that peptide **38** causes small breaches of the permeability barrier of the cytoplasmic membrane (or disruption of its proper functioning) resulting in rapid membrane depolarization. Membrane permeabilization appears after longer incubation times. Viability counts measured in parallel indicated that there is a good correlation between changes in membrane integrity and cell death. However, for *S. aureus*, no significant effect was observed on membrane potential (4%) or permeability (4%); whereas cell viability measured in parallel showed an 85% reduction. The differences between the results obtained by bacterial count and the PI staining indicate the presence of cells that maintained membrane integrity but were not able to grow, suggesting that the membrane is not the only target of compound **38** in *S. aureus.* Altogether, these results seem to suggest that different mechanisms of action may be involved in the activity of this lipopeptide, depending on the nature of the microorganism studied. The difference observed here between *S. aureus* and *E. coli* might be due to differences in envelope structure and composition. The cell walls of Gram-positive bacteria contain a peptidoglycan layer that is thicker than that in the cell walls of Gram-negative bacteria, providing the former bacteria with increased rigidity and this could be why no lysis of the membrane was barely observed in *S. aureus*, although other effects were seen by electron microscopy, as explained below. Another explanation could be that the lipopeptide also targets intracellular receptors, either a protein, nucleic acids or other potential targets, as has been observed with other AMPs^18^,^25^. PxB has been identified as an inhibitor of alternative NADH dehydrogenase^45^,^46^ and malate:quinone oxidoreductase in the Gram-positive *Mycobacterium smegmatis* (IC50 1.6 and 4.3 μg/mL, respectively). Kinetic analysis of inhibition by PxB has shown that the primary site of action is the quinone-binding site^45^.

### Transmission Electron Microscopy

**Figure 2:**
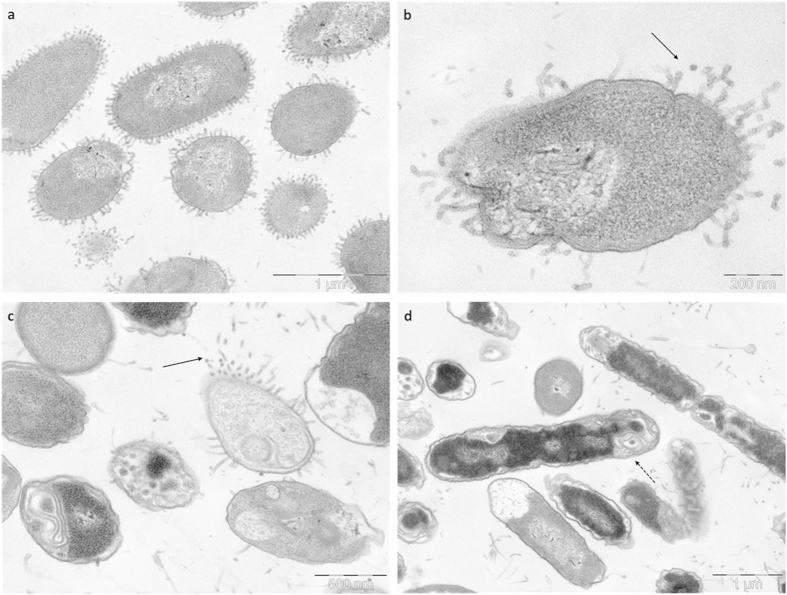
TEM micrographs of treated *E. coli.* **a,b**, after treatment with PxB (0.5 μg/mL) numerous protrusions (solid arrow) from the cell surface are observed. **c,d**, after treatment with lipopeptide **38** (2 μg/mL) the effect is diverse, showing numerous membranous structures, protrusions of the membrane similar to PxB (dashed arrow).

The effects of the lipopeptides studied on the cell morphology of *E. coli* and *S. aureus* were also observed via transmission electron microscopy (TEM). Untreated *E. coli* cells in a standard tryptone water medium showed a normal cell shape with an undamaged structure of the inner membrane and an intact outer membrane (data not shown). When *E. coli* was incubated with PxB as a control (120 min at 0.5 μg/mL: MIC), numerous protrusions were observed at the outer cell membrane and even some completely lysed membranes (Fig. 2a; solid arrow). Similarly, analog **38** induced notable modifications of the bacterial cell membranes, with different effects observed. The TEM images show abnormal membrane septation and the formation of blebs on the bacterial surface, similar to those induced by PxB (Fig. 2c, solid arrow). In addition, numerous intracellular membranous structures were observed in the polar cell regions together with a collapsed cytoplasm (Fig. 2d, dashed arrow). Moreover, the periplasmic space was highly swollen, probably due to the leakage of cellular material from the cytoplasm.

**Figure 3:**
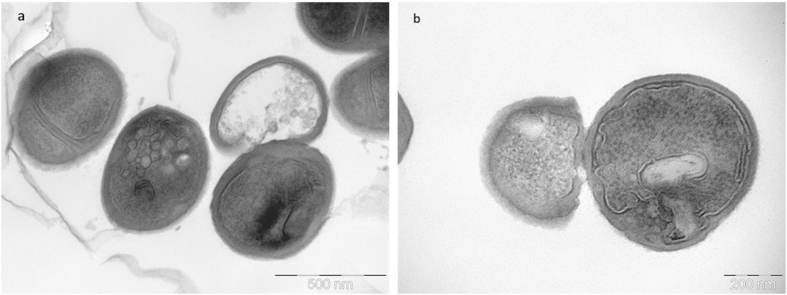
TEM micrographs of treated *S. aureus.* **a,b**, after treatment with lipopeptide **38** (4 μg/mL), mesosome-like structures and defects on the cell wall can be observed.

Treatment of *S. aureus* with lipopeptide **38** (at 4 μg/mL) induced numerous double-layered spherical mesosome-like structures as well as spherical non-membrane-enclosed bodies with an electron density similar to that of the septal cell wall layer (Fig. 3a). In addition, a retraction of the cytoplasm was observed. Mesosomes are intracytoplasmic membrane inclusions that indicate cytoplasmic membrane alteration caused by antibiotic activity. Since the cytoplasmic membrane is instrumental in cell wall synthesis and turnover, perturbation of this membrane may also affect cell wall integrity and autolysin regulation. Accordingly, the very fact that mesosome-like structures were seen in most treated cells indicates cytoplasmic membrane alteration and (possibly) uncoupling of the synthesis and turnover of cell wall polymers^47^. It has also been hypothesized that the formation of mesosomes in bacteria responds to a defensive mechanism to protect the bacteria from antibiotic assault. In fact, these invaginations have been observed after exposure to antibiotics as different as amikacin, gentamicin, ciprofloxacin, vancomycin, or oxacillin^48^.

## Conclusions

We designed and synthesized a series of cyclic lipopeptides based on a versatile and chemically accessible scaffold. The most active analogs were shown to have excellent antimicrobial activity against both Gram-negative (*P. aeruginosa* and *E. coli*) and Gram-positive (*S. aureus* and *E. faecalis*) clinically relevant bacteria. Their antimicrobial activity is at the membrane level although other targets may also be involved depending on the bacterial strain. Moreover, the most active sequence showed low toxicity *in vivo*, thus suggesting that a satisfactory therapeutic window could be achieved. Altogether, these new chemical entities are suitable compounds for hit-to-lead development. Synthesis of these novel cyclic lipopeptides can be readily scaled up for commercial use, including treatment of various infectious diseases caused by multi-drug-resistant bacteria.

## Experimental Part

### Peptide synthesis

Peptide synthesis was performed following standard Fmoc/^t^Bu procedures on Rink derivatised 4-methylbenzhydryl amine polystyrene resins, as described elsewhere^28^,^49^,^50^. Cyclization of peptides was carried out in DMSO:water (5:95) or ammonium bicarbonate buffer (100 mM, pH = 10) for 24–48 hours and monitored by analytical HPLC. The homogeneity was assessed by analytical HPLC using H_2_O-MeCN-TFA as eluants. Peptides were purified by semi-preparative HPLC to >95% and were characterized by analytical HPLC and MALDI-TOF or ESI mass spectrometries (see characterization of analogs **38** and **39** in the Supplementary Figure S5).

### *In vitro* microbiological activity in ATCC and resistant bacteria (MIC determination)

MICs were measured after 18–20 h of incubation in sterile 96-well plates (Corning Costar 3598 microtiter plates) in Mueller Hinton Broth (MHB) culture medium, (Difco, USA), according to well known *in vitro* protocols^51^. Bacterial strains in Table 1 were: *P. aeruginosa* ATTC 9027, *E. coli* ATTC 8739, *S. aureus* ATTC 6538, and *E. faecalis* ATTC 29212. MICs shown in table 4 were performed by Mary Conrad at Cubist Pharmaceuticals (Lexington, MA) in non-binding surface plates.

### *In vitro* cytotoxicity

Cell viability assays were performed using the tetrazolium dye WST1 (similar to MTT) on human dermal fibroblasts (3T3 hDF), MDCK (renal model, Madin-Darby canine kidney cells) and PC12 (NGF treated; neuronal model) cell lines and followed standard protocols^52^. Briefly, cells were seeded into 96-well plates, after incubation for 24 h, the medium was replaced with fresh cell culture medium containing the products (range 0-1.2 mg/mL). Negative and positive controls consisted of plates in the absence of the test compound and plates with sodium dodecyl sulfate (0.02% w/v), respectively. Cell viability was assayed with the tetrazolium salt (4-[3-(4- iodophenyl)-2-(4-nitrophenyl)-2H-5-tetrazolio]-1,3-benzene disulfonate) (WST-1). The relative WST-1 absorbance was recorded at 450 nm.

The products at concentrations ranging from 0 to 1.2 mg/mL were tested (triplicate) and in two independent experiments. The statistical analysis of the data was performed by using non-linear regression (curve-fit) and the model used agreed with the four parameter logistic model (4PL), also called the Hill-Slope model (sigmoid model), with the following equation:


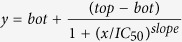


where y is the percent activity and x is the corresponding concentration. The quantities determined where the point estimate IC50 (Half maximal (50%) inhibitory concentration (IC) of a substance, see Table II), the corresponding 95% confidence interval and R^2^ (measures the goodness of fit) are shown in the Supplementary Table S2.

### *In vivo* toxicity studies

The evaluation of acute toxicity was conducted based on OECD 425 (Acute Oral Toxicity – Up-and-Down-Procedure (UDP) March 2006). The procedure was evaluated and accepted by the Ethical Committee of Experimental Animals of the University of Barcelona.

The main test consists of a single ordered dose progression in which animals are dosed, one at a time, at a minimum of 24-hour intervals.

Compound **38** was dissolved in PBS (pH 7.4), and administered subcutaneously to CD-1 mice at selected doses (100, 200 and 400 mg/kg). Since 5 reversals occurred in 6 consecutive animals tested between the 200 mg/Kg dose (the 3 animals, survived), and the 400 mg/Kg dose (the 3 animals died) the test was stopped.

Mortality was monitored for 14 days post-treatment, and LD50 was determined using the maximum likelihood method.

### Flow Cytometry

#### Bacteria, growth conditions and antimicrobial treatment

An overnight culture (12 to 16 h) of *S. aureus* (ATCC 29213) or *E. coli* (ATCC 25922) in Tryptic Soy Broth (TSB) was grown at 37 °C and 400 μL were added in 50 mL of tryptone water (∼10^7^ bacteria/mL). The bacterial suspension was split into aliquots of 4-5 mL and treated with the peptides (at the MIC) for different incubation times at room temperature. Cells were collected by centrifugation (10000 rpm, 20 min) and resuspended in 1 mL of filtered Ringer’s solution.

#### Staining procedure

A stock solution of propidium iodide (PI, 1 mg/mL, Molecular Probes) and bis-(1,3-dibutylbarbituric acid) trimethine oxonol (BOX, 250 μM, Sigma Aldrich) was prepared in distilled water. Portions (10 μL and 2 μL, respectively) were added to aliquots of cells (100 μL of each sample in 1 mL of Ringer’s solution) to give a final concentration of 10 μg/mL of PI and 0.5 μM for BOX.

#### Analysis

Flow cytometric measurements were performed on a Gallios multi-colour flow cytometer instrument (Beckman Coulter, Inc, Fullerton, CA) set up with the 3-lasers 10 colors standard configuration. Excitation was done using a blue (488 nm) laser. Forward scatter (FS), side scatter (SS), green fluorescence (525/40 nm) from BOX and red fluorescence (620/30 nm) emitted by PI were collected using logarithmic scales. FS was used as the discriminating parameter. The single-cell bacterial population was selected on a forward-side scatter plot. Fluorescence was presented in a dot plot of BOX vs. PI. Three regions were defined on this graph according to the controls: depolarized, permeabilized and non-affected cells. Control cells (non-stained and single stained populations) were used to define these regions. 25,000 cells defined according to their scatter parameters were counted in each sample.

### Transmission Electron microscopy (TEM)

#### Bacteria, growth conditions and antimicrobial treatment

An overnight culture (12 to 16 h) of *S. aureus* (ATCC 29213) or *E. coli* (ATCC 25922) in Tryptic Soy Broth (TSB) was grown at 37 °C and 500 μL were added in 50 mL of tryptone water (∼10^8^ bacteria/mL). The bacterial suspension was split into aliquots of 10 mL and treated with the peptides (at the MIC) for 120 minutes at room temperature. Cells were collected by centrifugation (10000 rpm, 20 min).

#### Transmission Electron microscopy preparation and observation

Samples were fixed with 2.5% glutaraldehyde in phosphate buffer for 2 h at 4 °C, then washed with the same buffer and post fixed with 1% osmium tetroxide in buffer containing 0.8% potassium ferricyanide at 4 °C. The samples were then dehydrated for 1 hour in acetone and infiltrated in a gradded series of Epon resin (Ted Pella Inc., USA) during 2 days, and finally embedded in fresh Epon resin and polymerised at 60 °C during 48 hours.

Ultrathin sections were obtained using a Leica Ultracut UCT ultramicrotome (Leica, Vienna) and mounted on Formvar-coated copper grids. Sections were stained with 2% aqueous uranyl acetate and lead citrate and examined in a JEM-1010 electron microscope (Jeol, Japan).

## Additional Information

**How to cite this article**: Rabanal, F. *et al.* A bioinspired peptide scaffold with high antibiotic activity and low *in vivo* toxicity. *Sci. Rep.* **5**, 10558; doi: 10.1038/srep10558 (2015).

## Supplementary Material

Supplementary Information

## Figures and Tables

**Table 1:**
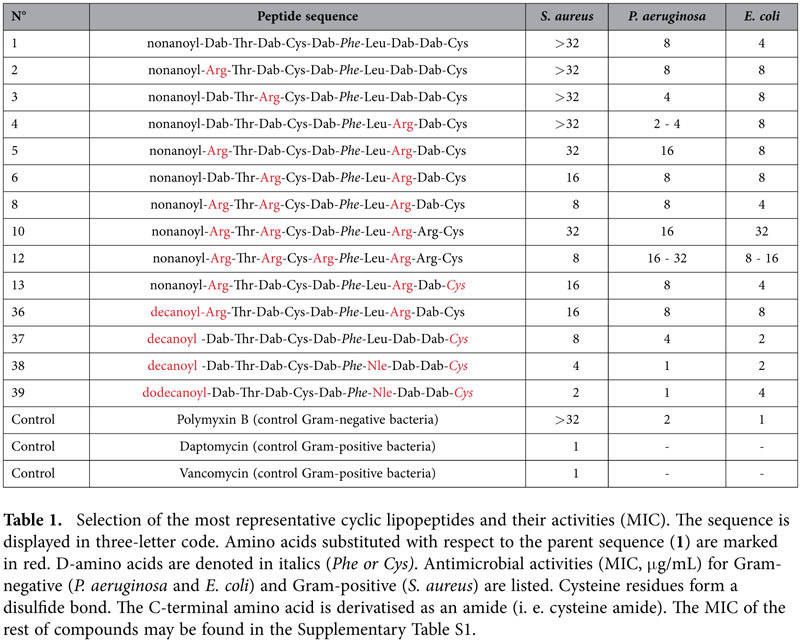
Selection of the most representative cyclic lipopeptides and their activities (MIC). The sequence is displayed in three-letter code. Amino acids substituted with respect to the parent sequence (**1**) are marked in red. D-amino acids are denoted in italics (*Phe or Cys)*. Antimicrobial activities (MIC, μg/mL) for Gram-negative (*P. aeruginosa* and *E. coli*) and Gram-positive (*S. aureus*) are listed. Cysteine residues form a disulfide bond. The C-terminal amino acid is derivatised as an amide (i. e. cysteine amide). The MIC of the rest of compounds may be found in the Supplementary Table S1.

**Table 2:** *In vitro* cytotoxicity of some lipopeptide analogs. IC50 (μg/mL) values of analogs 5, 8, and 12 having 2, 3 and 5 arginines, respectively, are shown. Cell cultures of human dermic fibroblasts (hDF), Madine-Darby canine kidney cells (MDCK, renal model) and PC12 (treated with NGF, neuronal model) have been used (see Supplementary Table S2 for statistics).

	**Analog 5 (2 Arg)**	**Analog 8 (3 Arg)**	**Analog 12 (5 Arg)**
hDF	534	461	164
MDCK	548	472	190
PC12	629	595	225

**Table 3:** Activity (MIC, μg/mL) of synthetic lipopeptide 39, daptomycin and vancomycin against Gram-positive *S. aureus* and *E. faecalis*, including clinical isolates and laboratory-derived daptomycin (DR) resistant strains from Cubist Pharmaceuticals (MS, methicillin sensitive; MR, methicillin resistant).

	***S. aureus***					***E. faecalis***
	**MS ATTC 29213**	**DR, MS SAU.278**	**MR ATTC 43300**	**MR SAU.1118**	**DR, MR SAU.1616**	**Wild type ATTC 29212**
**39**	1	2	1	1	8	2–4
daptomycin	0.5	8	0.5	0.5	16	2–4
vancomycin	0.5-1	2	2	0.5	2	2

**Table 4:** Activity (MIC, μg/mL) of lipopeptides 38 and 39 against resistant and multidrug-resistant Gram-negative *E. coli* and *P. aeruginosa* strains. MIC of colistin (polymyxin E) and polymyxin B are shown for comparison.

**Strain**	**Resistance profile**	**38**	**39**	**PxE**	**PxB**
*E. coli* MAC 21a	nalidixic acid, cotrimoxazole and ampicillin	0.5	1	0.5	0.25
*E. coli* VAL 10	nalidixic acid, cotrimoxazole and ampicillin	0.5	0.5	0.5	0.25
*E. coli* VAL 5	nalidixic acid	0.5	0.5	0.25	0.25
*E. coli* NDM-1	highly-resistant^a^	0.5	0.5	0.5	0.5
*P. aeruginosa* 40b	imipenem (MIC 32 mg/L)	4	2	1	2
*P. aeruginosa* 38a	ceftazidime, ciprofloxacin, imipenem, piperacillin-tazobactam	0.5	1	1	1

^a^Resistance profile (MIC): Amoxicillin 256 mg/L; Amoxicillin clavulanate 32 mg/L; Piperacillin/tazobactam 256 mg/L; Cefoxitin 256 mg/L; Cefotaxime 256 mg/L; Ceftazidime 256 mg/L; Cefepime 256 mg/L; Imipenem 8 mg/L; Meropenem 16 mg/L; Doripenem 6 mg/L; Ertapenem 24 mg/L; Aztreonam 256 mg/L; Gentamicin 8 mg/L; Amikacin 32 mg/L, Tobramycin 8 mg/L; Ciprofloxacin 32 mg/L (see^42^).

**Table 5:** Flow cytometry analysis and reduction of viability (obtained by plate count) of *E. coli* and *S. aureus* treated with PxB, PxBN and analog 38.

			***E. coli***			***S. aureus***		
			**% of stained cells**		**% viability reduction**	**% of stained cells**		**% viability reduction**
**Sample**	**Concentration (μg/mL)**	**Incubation time (min)**	**PI**	**BOX**		**PI**	**BOX**	
	-	30	3	5	-	1	5	-
Control	-	60	3	5	-	1	5	-
	-	120	2	4	-	2	5	-
	0.5	30	68	13	65	-	-	-
PxB	0.5	60	57	23	87	-	-	-
	0.5	120	71	17	98	-	-	-
PxBN	5	30	10	5	0	-	-	-
	5	60	3	6	5	-	-	-
	4	30	7	47	20	2	1	15
#**38**	4	60	23	50	60	3	1	60
	4	120	43	47	85	4	4	85
70 °C	-	30	80	11	100	35	25	100

## References

[b1] Morel C. M. & Mossialos E. Stoking the antibiotic pipeline. British Med. J. 340, 1115–1118 (2010). 10.1136/bmj.c2115 20483950

[b2] Cooper M. A. & Shlaes D. Fix the antibiotic pipeline. Nature 472, 32 (2011). 21475175 10.1038/472032a

[b3] Butler M. S., Blaskovich M. A. & Cooper M. A. Antibiotics in the clinical pipeline in 2013. J. Antibiotics 66, 571–591 (2013). 24002361 10.1038/ja.2013.86

[b4] Boucher H. W. *et al.* Infectious Diseases Society of America. 10×’20 Progress—Development of new drugs active against gram-negative bacilli: an update from the infectious diseases society of America. Clin. Infect. Dis. 56, 1685–1694 (2013). 23599308 10.1093/cid/cit152 PMC3707426

[b5] Guidos R. J., Infectious Diseases Society of America. Combating antimicrobial resistance: policy recommendations to save lives. Clin Infect Dis. 52, S397–S428 (2011). 21474585 10.1093/cid/cir153 PMC3738230

[b6] Mossialos E. *et al.* *Policies and Incentives for Promoting Innovation in Antibiotic Research*. (2009) Available at: http://www.lse.ac.uk/LSEHealthAndSocialCare/impacts/LSEHealthNews/News%20Attachments/Policies%20and%20incentives%20report.pdf. (Date of access: 04/02/2015)

[b7] CDC, Centre for Disease Control and Prevention, *Threat report 2013*. (2014) Available at: http://www.cdc.gov/drugresistance/threat-report-2013 (Date of access: 04/02/2015).

[b8] Smith R. & Coast J. The true cost of antimicrobial resistance. British Med. J. 346, f1430 (2013). 10.1136/bmj.f1493 23479660

[b9] Walsh C. T. & Wencewicz T. A. Prospects for new antibiotics: a molecule-centered perspective. J. Antibiotics 67, 7–22 (2014). 23756684 10.1038/ja.2013.49

[b10] World Health Organization. *Antimicrobial Resistance: Global Report on Surveillance 2014*. (2014) Available at: http://apps.who.int/iris/bitstream/10665/112642/1/9789241564748_eng.pdf (Date of access: 24/02/2015).

[b11] Woolhouse M. & Farrar J. An intergovernmental panel on antimicrobial resistance. Nature 509, 555–557 (2014). 24877180 10.1038/509555a

[b12] Editorial, A three-step plan for antibiotics, Nature 509, 533 (2014). 10.1038/509533a 24877183

[b13] Gilbert D. N. *et al.* The 10x´20 Initiative: pursuing a global commitment to develop 10 new antibacterial drugs by 2020. Clin. Infect. Dis. 50, 1081–1083 (2010). 20214473 10.1086/652237

[b14] Reardon S. Antibiotic resistance sweeping developing world. Nature 509, 141–142 (2014). 24805322 10.1038/509141a

[b15] Rex J. H. ND4BB: addressing the antimicrobial resistance crisis. Nature Rev. Microbiol. 12, 231–232 (2014).

[b16] Viñas M., Rabanal F., Benz R., Vinuesa T. & Fusté E. in Antimicrobial compounds: current strategies and new alternatives (eds Villa T. G. & Veiga-Crespo P. ) Ch. 10, 269–284 (Springer Verlag GmbH, 2014).

[b17] Fjell C. D., Hiss J. A., Hancock R. E. W., & Schneider G. Designing antimicrobial peptides: form follows function. Naure. Rev. Drug Discov. 11, 37–51 (2012). 10.1038/nrd3591 22173434

[b18] Pasupuleti M., Schmidtchen A. & Malmsten M. Antimicrobial peptides: key components of the innate immune system. Crit. Rev. Biotechnol. 32, 143–171 (2012). 22074402 10.3109/07388551.2011.594423

[b19] Yeaman M. R. & Yount N. Y. Mechanisms of antimicrobial peptide action and resistance. Pharmacol. Rev. 55, 27–55 (2003). 12615953 10.1124/pr.55.1.2

[b20] Yount N. Y. & Yeaman M. R. Emerging themes and therapeutic prospects for anti-infective peptides. Annu. Rev. Pharmacol. Toxicol. 52, 337–360 (2012). 22235859 10.1146/annurev-pharmtox-010611-134535

[b21] Brogden K. A. Antimicrobial peptides: Pore formers or metabolic inhibitors in bacteria? Nature Rev. Microbiol. 3, 238–250 (2005). 15703760 10.1038/nrmicro1098

[b22] Brogden N. K. & Brogden K. A. Will new generations of modified antimicrobial peptides improve their potential as pharmaceuticals? Int. J. Antimicrob. Agents 38, 217–225 (2011). 21733662 10.1016/j.ijantimicag.2011.05.004 PMC3159164

[b23] Srinivas N. *et al.* Peptidomimetic antibiotics target outer-membrane biogenesis in *Pseudomonas aeruginosa*. Science 327, 1010–1013 (2010). 20167788 10.1126/science.1182749

[b24] Finlay B. B. & Hancock R. E. W. Can innate immunity be enhanced to treat microbial infections? Nat. Rev. Microbiol. 2, 497–504 (2004). 15152205 10.1038/nrmicro908

[b25] Hancock R. E. W. & Scott M. G., The role of antimicrobial peptides in animal defences. Proc. Natl. Acad. Sci. USA 97, 8856–8861 (2000). 10922046 10.1073/pnas.97.16.8856 PMC34023

[b26] Rathinakumar R., Walkenhorst W. F. & Wimley W. C. Broad-spectrum antimicrobial peptides by rational combinatorial design and high-throughput screening: the importance of interfacial activity. J. Am. Chem. Soc. 131, 7609–7617 (2009). 19445503 10.1021/ja8093247 PMC2935846

[b27] Vaara M. Novel derivatives of polymyxins. J. Antimicrob Chemother. 68, 1213–1219 (2013). 23412346 10.1093/jac/dkt039

[b28] Rabanal F., Cajal Y., Garcia-Subirats M. & Rodríguez M., inventors; University of Barcelona assignee. Peptide compounds that can be used as antibacterial agents. United States patent US2013053305. 2010, March 10th

[b29] Nederberg F. *et al.* Biodegradable nanostructures with selective lysis of microbial membranes. Nature Chemistry 3, 409–414 (2011). 10.1038/nchem.1012 21505501

[b30] Hurdle J. G., O’Neill A. J., Chopra I. & Lee R. E. Targeting bacterial membrane function: an underexploited mechanism for treating persistent infections. Nature Rev. Microbiol. 9, 62–75 (2011). 21164535 10.1038/nrmicro2474 PMC3496266

[b31] Clark D. P., Durrel S., Malloy W. L. & Zasloff M. Ranalexin, a novel antimicrobial peptide from Bullfrog (*Rana Catesbeiana*) skin, structurally related to the antibacterial antibiotic polymyxin. J. Biol. Chem. 269, 10849–10855 (1994). 8144672

[b32] Rustici A. *et al.* Molecular mapping and detoxification of the lipid A binding site by synthetic peptides. Science 259, 361–364 (1993). 8420003 10.1126/science.8420003

[b33] Abdelraouf K., Jie H., Ledesma K. R., Hu M. & Tam V. H. Pharmacokinetics and renal disposition of polymyxin B in an animal model. Antimicrob. Agents Chemother. 56, 5724–5727(2012). 22908162 10.1128/AAC.01333-12 PMC3486600

[b34] Abdelraouf K., Chang K.-T., Yin T., Hu M. & Tam V. H. Uptake of polymyxin B into renal cells. Antimicrob. Agents Chemother. 58, 4200–4202 (2014). 24733472 10.1128/AAC.02557-14 PMC4068554

[b35] Yang J. J., Kularatne S. A., Chen X., Low P. S. & Wang E. Characterization of *in vivo* disulfide-reduction mediated drug release in mouse kidneys. Mol. Pharm. 9, 310–317 (2012). 22171616 10.1021/mp200483t

[b36] Yang J. J., Chen H., Vlahov I. R., Cheng J.-X. & Low P. S. Evaluation of disulfide reduction during receptor-mediated endocytosis by using FRET imaging, Proc. Natl. Acad. Sci. USA 103, 13872–13877 (2006). 16950881 10.1073/pnas.0601455103 PMC1564263

[b37] Geng Q., Sun X., Gong T. & Zhang Z.-R. Peptide–drug conjugate linked via a disulfide bond for kidney targeted drug delivery. Bioconjugate Chem. 23, 1200–1210 (2012). 10.1021/bc300020f 22663297

[b38] Velkov T., Thompson P. E., Nation R. L. & Li J. Structure-activity relationships of polymyxin antibiotics. J. Med. Chem. 53, 1898–1916 (2010) 19874036 10.1021/jm900999h PMC2907661

[b39] Chihara S., Yahata M., Tobita T. & Koyama Y. Chemical synthesis, isolation and characterization of α-N-fattyacyl colistin nonapeptide with special reference to the correlation between antimicrobial activity and carbon number of fatty acyl moiety. Agric. Biol. Chem. 38, 521–529 (1974).

[b40] Velkov T. *et al.* Teaching ‘Old’ Polymyxins New Tricks: New-Generation Lipopeptides Targeting Gram-Negative ‘Superbugs’. ACS Chem. Biol. 9, 1172–1177 (2014). 24601489 10.1021/cb500080r PMC4033650

[b41] Magee T. V. *et al.* Discovery of Dap-3 polymyxin analogues for the treatment of multi-drug-resistant gram-negative nosocomial infections. J Med Chem. 56, 5079–5093 (2013). 23735048 10.1021/jm400416u

[b42] Solé M. *et al.* First description of an *Escherichia coli* strain producing NDM-1 carbapenemase in Spain. Antimicrob. Agents Chemother. 55, 4402–4404 (2011). 21730115 10.1128/AAC.00642-11 PMC3165357

[b43] Alvarez-Barrientos A., Arroyo J., Canton R., Nombela C. & Sanchez-Perez M. Applications of flow cytometry to clinical microbiology. Clinical Microbiology Reviews 13, 167–195 (2000). 10755996 10.1128/cmr.13.2.167-195.2000 PMC100149

[b44] Comas J. & Vives-Rego J. Assessment of the effects of gramicidin, formaldehyde, and surfactants on *Escherichia coli* by flow cytometry using nucleic acid and membrane potential dyes. Cytometry 29, 58–64 (1997). 9298812 10.1002/(sici)1097-0320(19970901)29:1<58::aid-cyto6>3.0.co;2-9

[b45] Mogi T. *et al.* Polymyxin B identified as an inhibitor of alternative NADH dehydrogenase and malate:quinone oxidoreductase from Gram-positive bacterium *Mycobacterium smegmatis*. J. Biochem. 146, 491–499 (2009). 19564154 10.1093/jb/mvp096

[b46] Deris Z. Z. *et al.* A secondary mode of action of polymyxins against Gram-negative bacteria involves the inhibition of NADH-quinone oxidoreductase activity. J Antibiot. (Tokyo) 67, 147–151 (2014). 24169795 10.1038/ja.2013.111 PMC3943757

[b47] Friedrich C. L., Moyles D., Beveridge T. J. & Hancock R. E. W. Antibacterial action of structurally diverse cationic peptides on gram-positive bacteria. Antimicrob. Agents Chemother. 44, 2086–2092 (2000). 10898680 10.1128/aac.44.8.2086-2092.2000 PMC90018

[b48] Santhana R. L. *et al.* Mesosomes are a definite event in antibiotic-treated *Staphylococcus aureus* ATCC 25923. Tropical Biomedicine 24, 105–109 (2007). 17568383

[b49] Clausell A. *et al.* Gram negative outer and inner membrane models: insertion of cyclic cationic lipopeptides. J. Phys. Chem. B 111, 551–556 (2007). 17228913 10.1021/jp064757+

[b50] Clausell A., Rabanal F., Garcia-Subirats M., Alsina M. A. & Cajal Y. Membrane association and contact formation by a synthetic analogue of polymyxin B and its fluorescent derivatives. J Phys Chem B 110, 4465–4471 (2006). 16509750 10.1021/jp0551972

[b51] Jorgensen J. H. & Turnidge J. D. Antibacterial susceptibility test: dilutions and diffusion methods in Manual of Clinical Microbiology (eds Murray P. R., Baron E. J., Jorgensen J. H., Pfaller M. A. & Yorken R. H. ) pp 1108 (ASM Press: Washington DC, , 2003).

[b52] Mosmann T. Rapid colorimetric assay for cellular growth and survival: application to proliferation and cytotoxicity assays. J. Immunol. Methods 65, 55–63 (1983). 6606682 10.1016/0022-1759(83)90303-4

