# Supplementary material for: A bioinspired peptide scaffold with high antibiotic activity and low in vivo toxicity
Source: Sci Rep. 2015 May 29;5:10558. doi: 10.1038/srep10558 (PMC4603705; doi:10.1038/srep10558)
Supplement: Supplementary Information [file srep10558-s1.pdf]

Supplementary Information

A bioinspired peptide scaffold with high antibiotic activity and low *in vivo* toxicity

Francesc Rabanal, Ariadna Grau-Campistany, Xavier Vila-Farrés, Javier Gonzalez-Linares, Miquel Borràs, Jordi Vila, Mª Angels Manresa, Yolanda Cajal

[1]Department of Organic Chemistry, Faculty of Chemistry, University of Barcelona;

[2]Department of Physical Chemistry, Faculty of Pharmacy, University of Barcelona;

[3]Department of Microbiology, Faculty of Pharmacy, University of Barcelona;

[4]Barcelona Centre for International Health Research (CRESIB) Hospital Clínic – University of Barcelona;

[5]Experimental Toxicology and Ecotoxicology Unit /CERETOX, Barcelona Science Park.

Supplementary Table S1: MIC of additional lipopeptide analogs. Amino acids substituted with respect to the parent sequence (**1**) are marked in red. D-amino acids are denoted in italics (*Phe or Cys*). Antimicrobial activities (MIC, µg/mL) for Gram-negative (*P. aeruginosa* and *E. coli*, P. a. and E. c., respectively) and *S. aureus* (S. a.) are listed. Cysteine residues form a disulfide bond (underlined) except when specified as linear. C9 means nonanoyl moiety.

**Nº**	**Peptide sequence**	**S. a.**	**P. a.**	**E. c.**
7	C9-Arg-Thr-Arg-Cys-Dab-*Phe*-Leu-Dab-Dab-Cys	32	32	32
9	C9-Arg-Thr-Dab-Cys-Arg-*Phe*-Leu-Arg-Dab-Cys	16	128	16
11	C9-Arg-Thr-Arg-Cys-Arg-*Phe*-Leu-Arg-Dab-Cys	16	128	16
14	C9-Dap[(C=NH)NH_2]-Thr-Dab-Cys-Dab-*Phe*-Leu-Dap[(C=NH)NH_2]-Dab-Cys	16	16	2
15	C9-Dab[(C=NH)NH_2]-Thr-Dab-Cys-Dab-*Phe*-Leu-Dab[(C=NH)NH_2]-Dab-Cys	>32	8	4
16	C9-Arg(NO_2)-Thr-Dab-Cys-Dab-*Phe*-Leu-Arg(NO_2)-Dab-Cys	>32	16-32	4-8
17	C9-Arg-Thr-Dab-Cys-Dab-*Phe*-Leu-Arg-Dab-Cys Linear	32	16	64
18	C9-Arg-Thr-Dab-Ser-Dab-*Phe*-Leu-Arg-Dab-Ser Linear	64	64	32
19	C9-Dab-Thr-Dab-Cys-Dab-*Phe*-Met-Dab-Dab-Cys	32	16	4
20	C9-Dab-Thr-Dab-Cys-Dab-*Phe*-Met(O)-Dab-Dab-Cys	>32	8-16	>32
21	C9-Arg-Thr-Dab-Cys-Dab-*Phe*-Met-Arg-Dab-Cys	>32	8	8-16
22	C9-Arg-Thr-Arg-Cys-Dab-*Phe*-Met-Arg-Dab-Cys	32	16	16
23	C9-Arg-Thr-Dab-Cys-Dab-Phe-Leu-Arg-Dab-Cys	32	128	64
24	C9-Arg-Thr-Dab-Cys-Dab-Gly-Gly-Arg-Dab-Cys	>128	>128	>128
25	C9-Arg-Thr-Dab-Cys-Dab-Gly-Leu-Arg-Dab-Cys	>128	>128	>128
26	C9-Arg-Thr-Dab-Cys-Dab-*Phe*-Gly-Arg-Dab-Cys	128	>128	64
27	C9-Arg-Thr-Dab-Cys-Dab-*Phe*-Thr-Arg-Dab-Cys	64	8	16
28	C9-Arg-Thr-Dab-Cys-Dab-*Trp*-Leu-Arg-Dab-Cys	8	8	4
29	C9-Arg-Thr-Dab-Cys-Dab-*Leu*-Leu-Arg-Dab-Cys	32	16	32
30	C9-Arg-Thr-Dab-Cys-Dab-*Leu*-Thr-Arg-Dab-Cys	>128	32	32
31	C9-Arg-Thr-His-Cys-Dab-*Phe*-Leu-Arg-His-Cys	128	32	32
32	C9-Arg-Thr-Lys-Cys-Dab-*Phe*-Leu-Arg-Lys-Cys	32	16	8
33	C9-Lys-Thr-Dab-Cys-Dab-*Phe*-Leu-Lys-Dab-Cys	128	32	16
34	C9-Arg-Thr-Orn-Cys-Dab-*Phe*-Leu-Arg-Orn-Cys	32	16	8
35	C9-Orn-Thr-Dab-Cys-Dab-*Phe*-Leu-Orn-Dab-Cys	64	16	16

Supplementary Table S2: Statistical parameters of cell viability studies reported in Table 2. IC50 and confidence intervals are expressed in µg/mL.

			**Analog 5 (2 Arg)**	**Analog 8 (3 Arg)**	**Analog 12 (5 Arg)**
**hDF**	Exp1	IC50	555.2	420.8	147.0
		95% confidence int. –	497.6 –	398.8 –	141.6 –
		Goodness of Fit – R^2	0.9958	0.9948	0.9973
	Exp2	IC50	512.6	502.1	181.2
		95% confidence int. –	461.0 –	440.7 –	168.6 –
		Goodness of Fit – R^2	0.9913	0.9895	0.9922
**MDCK**	Exp1	IC50	540.9	416.8	232.6
		95% confidence int. –	495.4 –	394.6 –	223.2 –
		Goodness of Fit – R^2	0.9905	0.9943	0.9964
	Exp2	IC50	554.3	527.7	146.9
		95% confidence int. –	496.8 –	476.9 –	137.9 –
		Goodness of Fit – R^2	0.9905	0.9943	0.9964
**PC12**	Exp1	IC50	597.3	640.1	244.8
		95% confidence int. –	531.6 –	542.4 –	234.4 –
		Goodness of Fit – R^2	0.9955	0.9932	0.9966
	Exp2	IC50	660.3	550.2	205.9
		95% confidence int. –	620.9 –	514.8 –	196.9 –
		Goodness of Fit – R^2	0.9957	0.9956	0.9959

Supplementary Table S3: Flow cytometry analysis and reduction of viability (obtained by plate count) of *E. coli* treated with polymyxin B.

			E. coli		
			% of stained cells		% viability reduction
Sample	Concentration (µg/mL)	Incubation time (min)	PI	BOX	
PxB	0.5	5	11	45	0
	0.5	10	20	35	20
	0.5	15	60	17	34
	0.5	30	68	13	65
	0.5	60	57	23	87
	0.5	120	71	17	98

Supplementary Figure S4: Flow cytometry analysis of *E. coli* and *S. aureus* showing the effect of PxB and analog 38 on the membrane permeability (PI) and membrane potential (BOX). a,b, untreated control cells of *E. coli* and *S. aureus* respectively. c,d, *E. coli* cells after exposure to PxB and PxBN respectively. e,f, *E. coli* and *S. aureus* cells after exposure to analog 38. g,h, *E. coli* and *S. aureus* control of heated cells (70 ºC). In all cases, the incubation time was 2 hours except for heated cells (30 min).

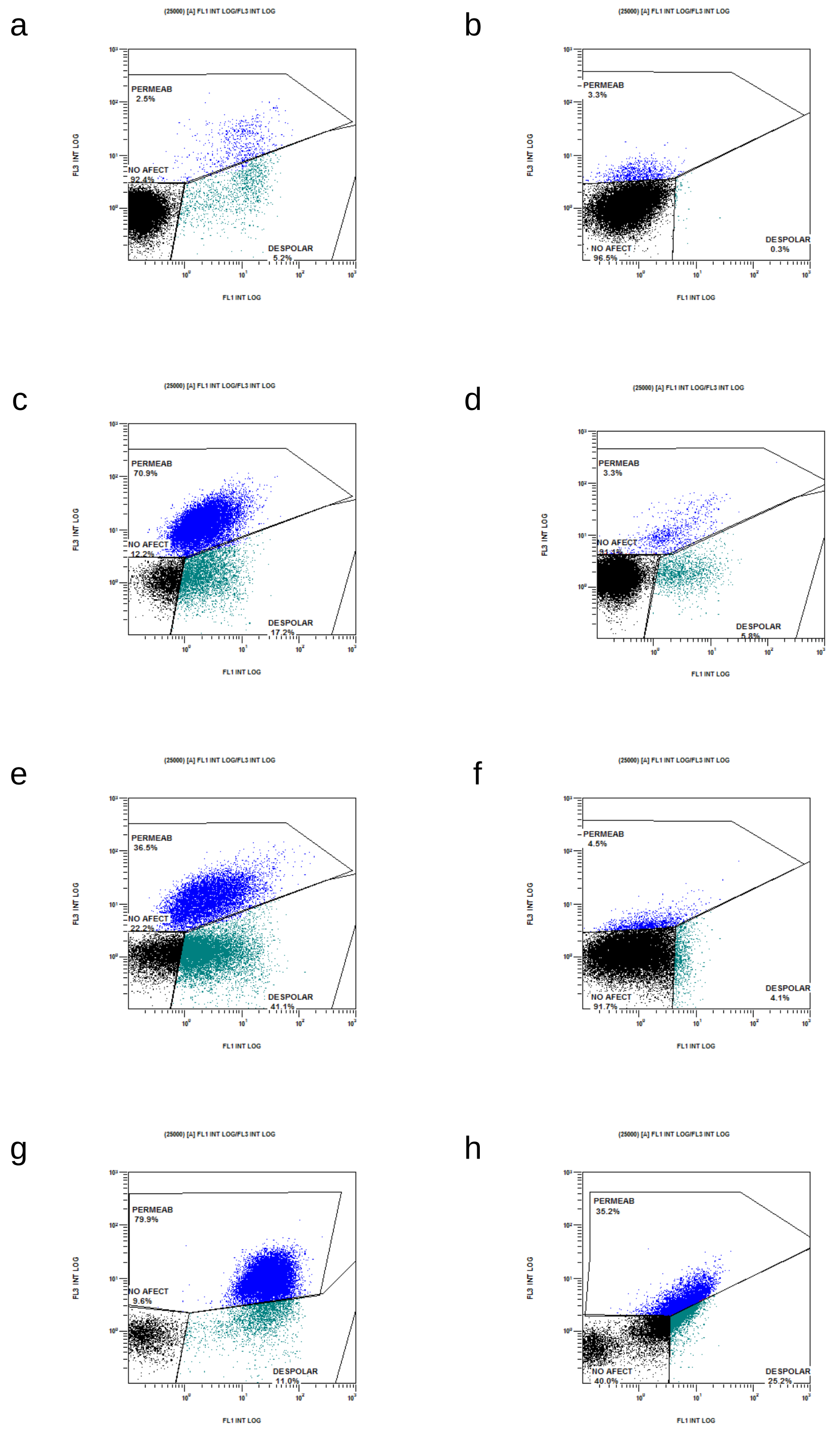

Supplementary Figure S5: Characterization by HPLC and MS of analogs **38** (upper) and **39** (lower). Elution conditions of 5% to 95% B in 30 min, solvent A: 0.045% TFA in H_2O and B: 0.036% TFA in acetonitrile (column: Nucleosil-C18, 120Å, 250x4.6 mm).

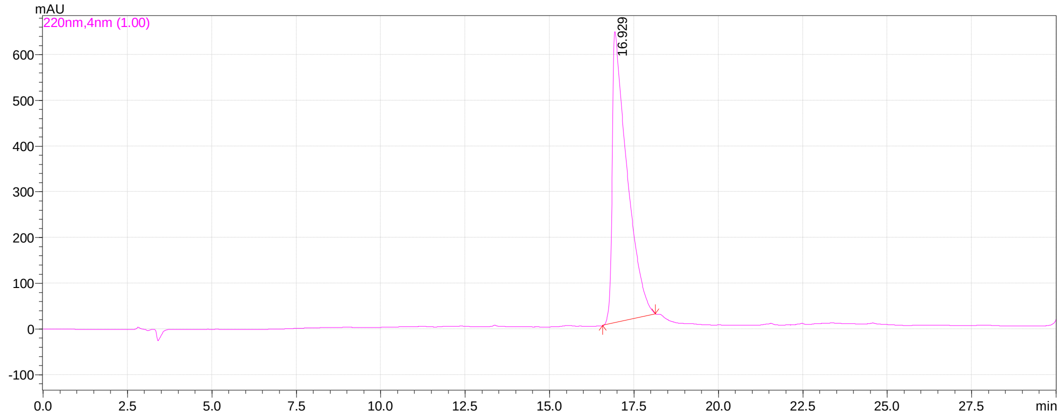

MALDI-TOF MS m/z ($C_{55}H_{96}N_{16}O_{12}S_2$): 1237.9 $[M+H]^+$, 1259.7$[M+Na]^+$, 1275.7 $[M+K]^+$, 1219.9 $[M-H_2O]^+$

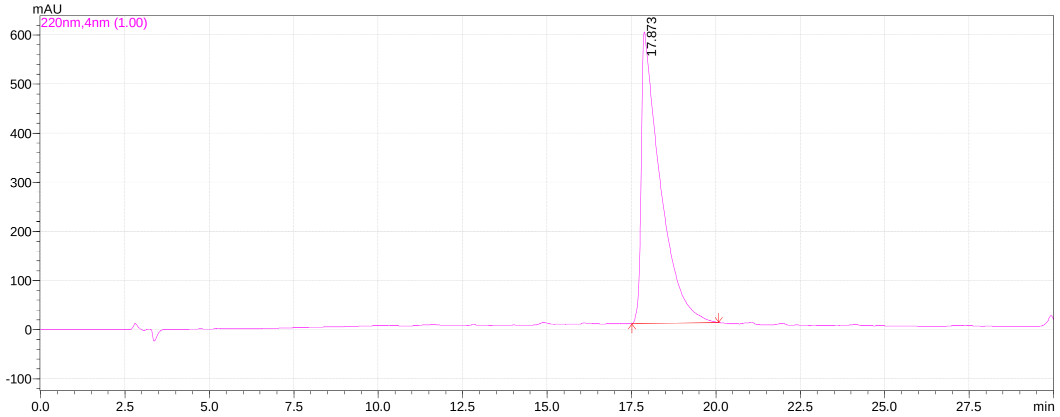

MALDI-TOF MS m/z ($C_{57}H_{100}N_{16}O_{12}S_2$): 1265.9 $[M+H]^+$, 1287.8 $[M+Na]^+$, 1303.7 $[M+K]^+$, 1247.8 $[M-H_2O]^+$
